# Seneca Valley virus 3C protease targets TRIM32 for cleavage to antagonize its antiviral effects

**DOI:** 10.1128/jvi.00590-25

**Published:** 2025-08-12

**Authors:** Jingjing Yang, Zijian Li, Ruiyi Ma, Shijie Xie, Dan Wang, Rong Quan, Jue Liu, Jiangwei Song

**Affiliations:** 1 Beijing Key Laboratory for Prevention and Control of Infectious Diseases in Livestock and Poultry, Institute of Animal Husbandry and Veterinary Medicine, Beijing Academy of Agriculture and Forestry Sciences 656308 , Beijing, China; 2 College of Animal Science and Veterinary Medicine, Shenyang Agricultural University 98428, Shenyang, China; 3 College of Veterinary Medicine, Shanxi Agricultural University 74600, Taigu, Shanxi, China; 4 College of Veterinary Medicine, Yangzhou University 38043, Yangzhou, China; University of Michigan Medical School, Ann Arbor, Michigan, USA

**Keywords:** Seneca Valley virus (SVV), TRIM32, 3C protease, cleavage, type I interferon (IFN-I)

## Abstract

The Seneca Valley virus (SVV), a newly emerged virus within the *Picornaviridae* family, causes porcine idiopathic vesicular disease, imposing substantial economic costs to the global pork industry. However, the molecular mechanisms underlying its pathogenesis remain poorly understood. In this study, we identified TRIM32 as a novel antiviral factor against SVV and found that SVV infection led to the cleavage and degradation of TRIM32. Knocking down TRIM32 increased viral replication, while overexpressing it reduced viral titers. Further study showed that TRIM32 restricts SVV replication by selectively targeting viral VP3 protein for ubiquitination and proteasomal degradation. To counteract this, SVV 3C protease (3Cpro) targets TRIM32 for cleavage at E332. This cleavage renders TRIM32 unable to inhibit SVV replication or block the degradation of VP3. Additionally, the cleaved TRIM32 products weaken E3 ubiquitin ligase activity and reduce activation of the type I interferon (IFN) pathway. Taken together, our results indicate that SVV 3C^pro^-mediated cleavage of TRIM32 impairs its function in the ubiquitination and degradation of viral VP3 and type I IFN signaling. These findings offer novel insights into the strategies viruses use to evade the host’s antiviral immune responses, thereby contributing to efficient viral replication.

**IMPORTANCE:**

Seneca Valley virus (SVV) is an emerging pathogen that causes vesicular diseases in pigs, posing a significant threat to the global swine industry. Tripartite motif-containing protein (TRIM) family members are recognized as intrinsic antiviral effectors that provide a frontline shield against viruses prior to the transcription of interferon (IFN) and interferon-stimulated genes (ISGs). In this study, we uncovered the antiviral mechanism, which promotes the K48-linked ubiquitination of viral VP3 protein, leading to the degradation of VP3 via the proteasome pathway. SVV 3C^pro^ abolished the antiviral effects of TRIM32 by inducing its cleavage. The cleaved TRIM32 fragment attenuates its E3 ubiquitin ligase activity and weakens the activation of IFN signaling. Our results reveal a potential mechanism of viral immune evasion, which is crucial for understanding how SVV has evolved a novel strategy to evade the intrinsic cellular restrictions against viral infection.

## INTRODUCTION

Seneca Valley virus (SVV), also known as Senecavirus A (SVA), is a non-enveloped virus with a positive-sense, single-stranded RNA virus that belongs to the *Senecavirus* genus within the *Picornaviridae* family (1). The first recognized strain of SVV was initially discovered as a contaminant in PER.C6 cell cultures. It was first identified and isolated in the United States in 2002 and was initially used as an oncolytic virus for cancer treatment (2, 3). SVV infection can cause vesicular diseases in pigs, and its clinical manifestations closely resemble those of foot-and-mouth disease (FMD) and other porcine vesicular diseases (4).

SVV possesses a single open reading frame (ORF) that encodes a large polyprotein that is subsequently processed into structural and nonstructural proteins, including leader protein (Lpro), P1 (VP4-VP2-VP3-VP1), P2 (2A-2B-2C), and P3 (3A-3B-3C^pro^-3D^pol^) (1). Initially, the structural proteins VP0, VP1, and VP3 form pentamers of SVV particles (1). Subsequently, the precursor VP0 is further cleaved into VP2 and VP4 to assemble the complete capsids (1, 5). The coat-shell proteins VP1 and VP2 interact with VP3 and the cell receptor ANTXR1, and other potential cell receptors, thereby initiating infection (6).

SVV has evolved multiple strategies to evade the host’s innate immune response. For instance, SVV 3C protease (3C^pro^) suppresses the production of type I interferon (IFN) by degrading and cleaving numerous critical molecules in the innate immune pathway (7–10). SVV 3C^pro^ targets key innate immune adapters such as the mitochondrial antiviral-signaling protein (MAVS), TIR-domain-containing adapter-inducing IFN-β (TRIF), and TRAF family member-associated NF-κB activator (TANK) for cleavage, resulting in cleaved MAVS and TANK products that fail to regulate IFN-mediated responses (7). 3C^pro^ also cleaves porcine cyclic GMP-AMP synthase (cGAS), disrupting cGAS-mediated DNA binding, cGAMP synthesis, and IFN induction (8). Moreover, 3C^pro^ cleaves the transducer and activator of transcription 2 (STAT2), interfering with its nuclear import and formation of the IFN-stimulated gene factor 3 complex (ISGF3) to escape host immunity (10). Additionally, 3C^pro^ inhibits the ubiquitination of retinoic acid-inducible gene I (RIG-I), TANK-binding kinase 1 (TBK1), and tumor necrosis factor receptor-associated factor 3 (TRAF3), blocking the expression of mRNA of IFN-β and IFN-stimulated gene 54 (ISG54) (9). SVV 3C^pro^ also targets the adaptor protein, optineurin (OPTN), for cleavage, impeding selective autophagy and type I IFN production (11). Furthermore, it cleaves the selective autophagy receptor, SQSTM1/p62 (sequestosome 1), to evade the host autophagic machinery for survival (12).

Tripartite motif-containing proteins (TRIMs) are a family consisting of approximately 100 members in humans. This family is a highly conserved superfamily of proteins composed of a conserved N-terminal RING domain, followed by B-box, coiled-coil, and C-terminal domains (13, 14). The RING domain is crucial for its E3 ubiquitin ligase activity because the B-box domain does not promote enzyme activity and is involved in protein assembly and interaction. Therefore, the coiled-coil domain is necessary for protein dimerization, and the C-terminal domain is diverse and forms complex proteins with complex biological functions (15, 16). TRIM family members are versatile proteins known for their antiviral activities against various viral infections. They act either by directly targeting viral components or by regulating innate immune responses (17–19). For example, Coxsackievirus B3 (CVB3) 3C^pro^ cleaves TRIM7, weakening its antiviral capacity as the cleaved TRIM7 dampens its E3 ubiquitin ligase activity (20). TRIM7 restricts multiple enteroviruses by targeting viral 2BC for ubiquitination and proteasome-dependent degradation (21). Similarly, TRIM22 ubiquitinates the hepatitis C virus (HCV) NS5A protein to inhibit HCV replication (22). In contrast, TRIM79α restricts tick-borne encephalitis virus replication by mediating lysosome-dependent degradation of viral NS5 protein (23). TRIM43 targets the centrosomal protein, pericentrin, for proteasomal degradation, altering nuclear membrane integrity and repressing active viral chromatin states (24). In addition, TRIM21 and TRIM23 exert antiviral activity against fish viruses by positively regulating the host IFN response (25, 26).

TRIM32, initially identified as a binding partner of the retroviral tat protein (27), is a multifunctional E3 ubiquitin ligase involved in diverse cellular processes including innate immunity, development, differentiation, and antiviral immunity (28). Structurally, this protein features a conserved N-terminal RING-finger domain followed by a B-box and coiled-coil domain, and a series of NHL repeats. The RING domain confers E3 ubiquitin ligase activity critical for its functional roles. TRIM32 senses and restricts the influenza A virus (IAV) by targeting polymerase subunit 1 (PB1) for ubiquitination and protein degradation (29). In addition, TRIM32 participates in the STING-mediated type I IFN pathway and exhibits antiviral effects against influenza viral infections (30). Overexpression of TRIM32 positively regulates the IFN immune response, whereas deletion of its RING domain abolishes both its antiviral activity and regulatory roles in IFN-mediated immunity and inflammatory responses (31). Mechanistically, TRIM32 exerts its antiviral function through direct ubiquitination of STING (also known as MITA), resulting in robust induction of IFN-β transcription and subsequent augmentation of antiviral effector responses (32). However, the mechanism through which the TRIM family is involved in SVV replication remains unclear.

In this study, we found that TRIM32 binds to SVV VP3 and promotes its degradation via the proteasome pathway to inhibit viral replication. Mechanistically, SVV 3C^pro^ specifically cleaves TRIM32 at E332, which dramatically attenuates the antiviral effects of TRIM32 by weakening its E3 ubiquitin ligase activity and IFN response. Specifically, the cleaved TRIM32 products lose the ability to degrade VP3 and inhibit SVV replication. In summary, our findings reveal the diverse strategies SVV uses to evade the host antiviral immune response and provide a theoretical basis for the development of new antiviral strategies.

## MATERIALS AND METHODS

### Cells, viruses, antibodies, and reagents

BHK-21 (baby hamster kidney-21) cells, HEK-293T (human embryonic kidney-293T) cells, PK-15 (porcine kidney-15) cells, HeLa cells, and mouse myoblast cell line (C2C12 cells) were maintained in Dulbecco’s modified Eagle’s medium (DMEM) (Gibco, USA) supplemented with 10% fetal bovine serum (FBS; Gibco). The SVV strain CHhb17 (GenBank: MG983756.1) and porcine epidemic diarrhea virus (PEDV) strain LZW (GenBank: KJ777678.1) used in this study as previously described (33, 34). Pseudorabies virus (PRV) strain SX1910 (GenBank: OL606749.1) and anti-PRV VP5 antibody used in this study were graciously provided by Dr. Jianle Ren (Shanxi Agricultural University) as previously described (35). HSV-1 (herpes simplex virus type 1) F strain was provided by Prof. Chunfu Zheng (University of Calgary) as previously described (36). The eGFP-inserted rescued Seneca Valley virus (rSVV-eGFP) was graciously provided by Dr. Fuxiao Liu (Qingdao Agricultural University) (37). The GFP rabbit polyclonal antibody (50430-2-AP) was purchased from Proteintech (Wuhan, China). The GFP mouse monoclonal antibody (ab127417) and β-actin mouse monoclonal antibody (ab8226) were purchased from Abcam (Cambridge, MA, USA). HA monoclonal antibody (H3663) was obtained from Sigma-Aldrich (St. Louis, MO, USA). Rabbit anti-HA (3724), rabbit anti-STING (13647), and rabbit anti-TBK1 (3504) were purchased from Cell Signaling Technology (Beverly, MA, USA). SVV VP1 mouse monoclonal antibody was used in this study as previously described (33). SVV VP3 polyclonal antibody and PEDV N polyclonal antibody were prepared in our lab. Alexa-568-conjugated goat anti-rabbit secondary antibody (A11011), Alexa-488-conjugated goat anti-mouse secondary antibody (A10680), and Alexa-647-conjugated goat anti-mouse secondary antibody (A32728) were obtained from Invitrogen. MG132 (proteasome inhibitor, S2619), Z-VAD-FMK (caspase inhibitor, S7023), bafilomycin A1 (autophagy inhibitor, Baf A1, S1413), chloroquine (autophagy inhibitor, CQ, S6999), and 3-methyladenine (autophagy inhibitor, 3-MA, S2767) were purchased from Selleck Chemicals (Shanghai, China).

### Plasmid construction

TRIM32 gene was cloned from PK-15 cells and reconstructed into pCMV-HA vector and pCMV-myc vector (Clontech, 631604) by using one-step DNA assembly kit (D0204P, Lablead). TRIM32 gene with site mutation was generated by site-directed mutagenesis using KOD DNA polymerase (TOYOBO, KFX-201). A series of GFP-tagged encoding SVV structural and nonstructural protein expression plasmids, GFP-3C carrying single mutation (H48A or C160A) and double mutation (H48A and C160A) that have been used in our previous studies (10, 11). The 3C sequence of EMCV (GenBank: KF836389.1), FMDV (GenBank: AF088223.1), Rhinovirus (GenBank: MN228695.1), CVB3 (GenBank: KX981987.1), and EV71 (GenBank: KT428649.1) was synthesized from Tsingke Biotechnology (Beijing, China) and recombined into the vector pEGFP-C1 that has been used in our previous studies (10, 11). Flag-STING (GenBank: MF622062.1), Flag-MDA5 (GenBank: AF095844.1), and Flag-MAVS (GenBank: NM_020746.5) were synthesized from RuiBiotech Biotechnology Co., Ltd. (Beijing, China). myc-STING and its truncation construct were subcloned from Flag-STING. HA-ubiquitin has been used in our previous study (11). The primers used are listed in Table 1.

**TABLE 1:** Primers used in this study

Primer^*a*^	Sequence (5′−3′)
HA-myc-TRIM32-EcoRI-F	TGGCCATGGAGGCCCGAATTCGGATGAGCAGGACTTGGCCCTCTGGA
HA-myc-TRIM32-KpnI-R	GATCCCCGCGGCCGCGGTACCCTAAGGGGTAGAATATCTTCTCAG
Human IFN-β-F	TTGCTCTCCTGTTGTGCTTC
Human IFN-β-R	AAGCCTCCCATTCAATTGCC
Human ISG56-F	CCTCCTTGGGTTCGTCTACA
Human ISG56-R	GGCTGATATCTGGGTGCCTA
Human OAS1-F	GACGATGAGACCGACGATCC
Human OAS1-R	GTGCAGGTCCAGTCCTCTTC
Human GAPDH-F	GAGTCAACGGATTTGGTCGT
Human GAPDH-R	GACAAGCTTCCCGTTCTCAG

^*a*^ F denotes forward PCR primer; R denotes reverse PCR primer.

### Western blot

Cells were harvested using cell lysis solution (0.5 mM EDTA, 10 mM Tris, pH 7.5, 150 mM NaCl, and 0.5% NP-40) for 30 min at 4°C with rotation. The whole cell lysates were clarified by centrifugation at 13,000 rpm for 15 min. The clarified lysates were then separated by 10% sodium dodecyl sulfate-polyacrylamide gel electrophoresis (SDS-PAGE), and it was blotted onto a nitrocellulose membrane (66485, PALL). Phosphate-buffered saline (PBS) with 5% skimmed milk powder (BS102, Biosharp, China) blocked the membrane for 1 h at room temperature (RT). Incubate membrane with primary antibody overnight at 4°C. Afterward, thoroughly wash the membrane with wash buffer 0.01 M PBST (PBS + 0.1% tween-20). Membrane was incubated with horseradish peroxidase (HRP)-conjugated goat anti-mouse IgG (1706516, Bio-Rad) or HRP goat anti-rabbit IgG (1706515, Bio-Rad) for 1 h (RT). Afterward, thoroughly wash the membrane with PBST and detect the blots with a chemical luminescent substrate (E1070, Lablead, China).

### Co-immunoprecipitation (Co-IP)

The cells were harvested using a cell lysis solution (0.5 mM EDTA, 10 mM Tris, pH 7.5, 150 mM NaCl, and 0.5% NP-40) for 30 min at 4°C with rotation. The whole cell lysates were clarified by centrifugation at 13,000 rpm for 15 min. The cell supernatants were mixed with anti-HA magnetic beads (HY-K0201, MedChemExpress, China) on a rotary mixer overnight. The beads were washed three times with lysis buffer and eluted with 1 × SDS loading buffer at 100°C, then subjected to SDS-PAGE for Western blot analysis.

### *In vitr*o cleavage assay

Flag-TRIM32-WT and Flag-TRIM32-E332 were purified from BHK-21 cells using anti-Flag resin (B26102, Selleck) and eluted by Flag–peptide (RP10586CN, Genscript). The elution buffer was prepared by diluting Flag-peptide in buffer containing 100 mM Tris–HCl (pH 7.5), 150 mM NaCl, and 1 mM dithiothreitol (DTT) at a final concentration of 200 ng/mL. His-3C-WT and His-3C-DM were expressed and purified from *Escherichia coli*. Flag-TRIM32-WT (5 µg) and Flag-TRIM32-E332 (5 µg) were incubated with His-3C-WT (5 and 10 µg) or His-3C-DM (10 µg) in a 25 µL reaction containing 50 mM HEPES (pH 7.5), 3 mM EDTA, 150 mM NaCl, 0.005% (vol/vol) Tween-20, and 10 mM DTT at 37°C for 2 h, followed by Western blot.

### Quantitative RT-PCR

Total RNA was extracted from cells using TRIeasy Total RNA Kit (19221ES50, Yeasen, China). The first strand cDNA was synthesized using 1 µg total RNA with SYBR Green-based quantitative real-time PCR (F0202A, Lablead, China). Each experimental sample was replicated three times. The amplification cycling conditions were set as follows: 94°C for 5 min, followed by 45 cycles of 5 s at 94°C, 10 s at 60°C, and 15 s at 72°C. GAPDH was employed as a control gene. The data were calculated using the 2^−ΔΔCT^ method, and significant differences among the data were analyzed. The primers used are listed in Table 1.

### Indirect immunofluorescence

Cells were fixed with 4% paraformaldehyde for 10 min (RT). Subsequently, the samples were permeabilized using a solution of 2% bovine serum albumin (BSA) in PBS containing 0.1% Triton X - 100 for 10 min. Blocking the cells with 2% BSA in PBS for 30 min (RT), then incubating with primary antibody for 2 h (RT). After washing with PBS, the corresponding fluorescent secondary antibodies Alexa-488-labeled goat anti-mouse IgG (H + L) (11017, Invitrogen) and Alexa-568-labeled goat anti-rabbit IgG (H + L) (11011, Invitrogen) were diluted (1:1000) in PBS and incubated with the cells for 1 h (RT). The nuclei were stained with DAPI (4',6-diamidino-2-phenylindole). Images were taken under a Nikon A1 confocal microscope (Tokyo, Japan).

### RNA interference (RNAi)

The small interfering RNAs (siRNAs) targeting the TRIM32 and siNC gene (control group) were synthesized by GenePharma (Suzhou, China). The siRNAs target sequences were: si-TRIM32 (sense, 5′-GCAGCUGCGUCCCAAGCUUTT-3′; antisense, 5′-AAGCUUGGGACGCAGCUGCTT-3′); si-STING (sense, 5′-CCCAGUACCUCCACGAUGUTT-3′; antisense, 5′-ACAUCGUGGAGGUACUGGGTT-3′); si-TBK1 (sense, 5′-GCAGAGCACUUCUAAUCAUTT-3′; antisense, 5′-AUGAUUAGAAGUGCUCUGCTT-3′); siNC (sense, 5′-UUCUCCGAACGUGUCACGUTT-3′; antisense, 5′-ACGUGACACGUUCGGAGAATT-3′) used as a negative control. The siRNAs were transfected into cells at a final concentration of 20 pmol using RNAiMAX transfection reagent (13778150, Thermo Fisher).

### TCID_50_ assay

The cells seeded in six-well tissue culture plates were washed with PBS and inoculated with SVV at an indicated multiplicity of infection (MOI). The culture was incubated at 37°C for 1 h. The infected cells were washed three times with PBS to remove unbound virus particles and covered with DMEM containing 2% FBS. The infected cells were incubated at 37°C and harvested at the indicated times. After three consecutive freeze-thaw cycles, the virus titer was determined by TCID_50_ assay.

### TRIM32 knockout cell line construction

To generate TRIM32 knockout C2C12 cells, two TRIM32 targeting sgRNA (sgRNA1: GATACGATAGTTGCCCCGGT; sgRNA2: CATCTCTCTAAAGGTTACCG) were cloned into LentiCRISPRv2-Puro and LentiCRISPRv2-Blast vector (gifts from Brett Stringer, Addgene plasmids: #98290 and #98293), respectively. The lentivirus was prepared as previously described (38). C2C12 cells were transduced with the lentiCRISPRv2-Puro and lentiCRISPRv2-blast derived lentivirus together for 48 h, then replated into complete DMEM containing 4 µg/mL puromycin and 15 µg/mL blasticidin for 3 days selection process to eliminate the non-transduced cells. The knockout efficiency was evaluated by Western blot using specific antibody against TRIM32.

### Flow cytometry

The proportion of GFP-positive cells was determined by means of an ACEA NovoCyte flow cytometer (ACEA Biosciences, CA, USA) within the FITC channel. In total, 10,000 single-cell events were selected through flow cytometry.

### Statistical analysis

Data are presented as the means ± standard deviations (SDs) and were analyzed using GraphPad Prism software. *P* values of <0.05 were considered statistically significant.

## RESULTS

### SVV infection induces TRIM32 degradation and cleavage

**Fig 1:**
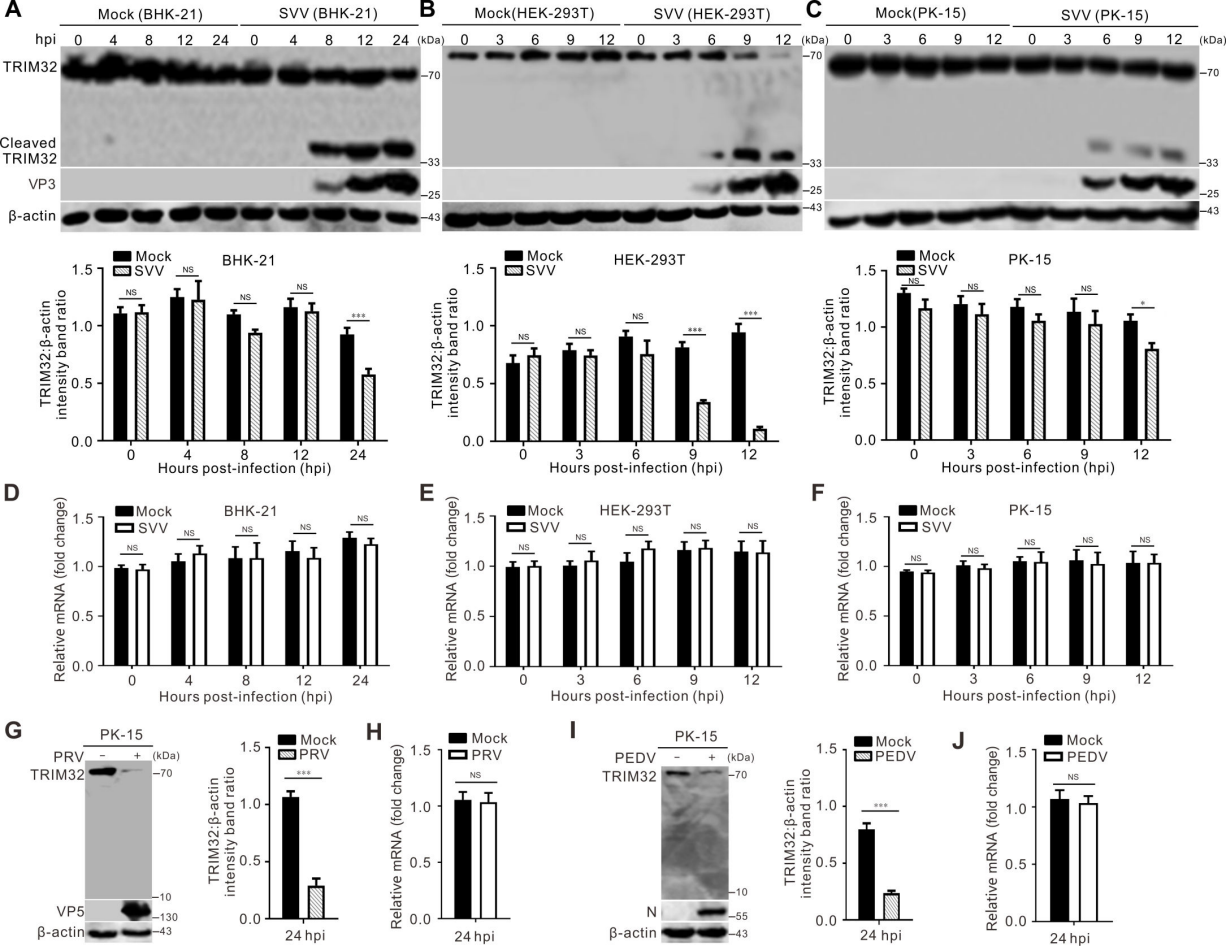
SVV infection induces cleavage and degradation of TRIM32. (A–C) BHK-21 cells (**A**), HEK-293T cells (**B**), and PK-15 cells (**C**) seeded in six-well plates were infected with SVV at a multiplicity of infection (MOI) of 5. Cells were collected at indicated times, and the expression levels of endogenous TRIM32 protein were detected by Western blot. Quantification of TRIM32 expression from figure (**A–C**) was performed using ImageJ, the level of full-length TRIM32 band was normalized to that of β-actin, respectively. Data are presented as the mean ± standard deviation (SD) derived from three independent experiments (*, *P* < 0.05; ***, *P* < 0.001; NS, not significant). (**D–F**) BHK-21 cells (**D**), HEK-293T cells (**E**), and PK-15 cells (**F**) in six-well plates were infected with SVV (MOI = 5), the cells were harvested at indicated times, the TRIM32 mRNA levels were determined by real-time quantitative polymerase chain reaction (RT-PCR). (**G, I**) PK-15 cells seeded in six-well plates were infected with PRV (MOI = 0.1) and PEDV (MOI = 0.1) for 24 h, respectively. The expression levels of endogenous TRIM32 protein were detected by Western blot. Quantification of TRIM32 expression from figure (**G, I**) was performed using ImageJ, and the level of TRIM32 band was normalized to that of β-actin, respectively. Data are presented as the mean ± SD derived from three independent experiments (***, *P* < 0.001; NS, not significant). (**H, J**) PK-15 cells seeded in six-well plates were infected with PRV (MOI = 0.1) and PEDV (MOI = 0.1) for 24 h, respectively. The TRIM32 mRNA levels from figure (**H, J**) were determined by real-time quantitative RT-PCR.

SVV infection induced both degradation and cleavage of TRIM32 in BHK-21, HEK-293T, and PK-15 cells, generating two proteolytic products with a TRIM32 antibody (Fig. 1A through C). In addition, TRIM32 was degraded throughout the SVV infection (Fig. 1A through C), while TRIM32 was not cleaved or degraded in mock-infected cells (Fig. 1A through C). Real-time quantitative polymerase chain reaction (RT-qPCR) revealed that the transcriptional level of TRIM32 did not respond to SVV infection, indicating that the decreased abundance of TRIM32 protein was not involved at the transcriptional level over the course of infection (Fig. 1D through F). PEDV and PRV were used to evaluate the changes in TRIM32 to exclude the factors not specific to SVV infection induced cell death, immune responses, and so on. As shown in Fig. 1G through J, the infection of PEDV and PRV led to the degradation of TRIM32, while the transcriptional level of TRIM32 remained unaffected. These results suggest that SVV infection cleaves and degrades TRIM32 in host cells.

### TRIM32 inhibits SVV proliferation

**Fig 2:**
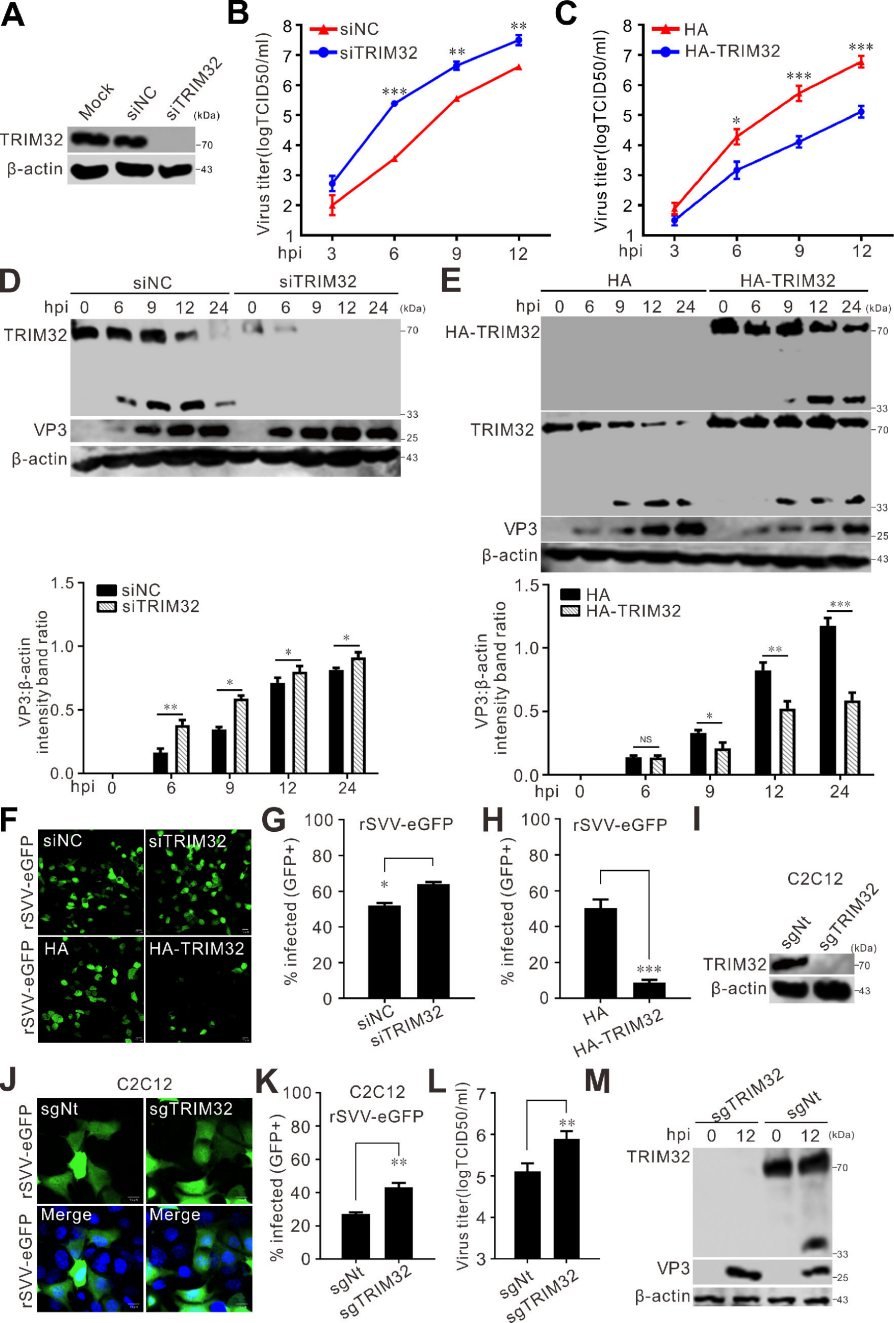
TRIM32 inhibits SVV replication. (**A**) HEK-293T cells seeded in six-well plates and transfected with siTRIM32 and siNC (negative control) each at a concentration of 20 pmol. At 36 h post-transfection (hpt), the cells were lysed and subjected to Western blot. (**B, C**) HEK-293T cells seeded in six-well plates were transfected with indicated siRNAs (**B**) and plasmids (**C**) for 36 h. Then, the cells were infected with SVV (MOI = 0.5). The virus titers in the supernatants at indicated time were determined using the TCID_50_ assay. (**D, E**) HEK-293T cells seeded in six-well plates were transfected with indicated siRNAs (**D**) and plasmids (**E**). At 36 hpt, the cells were infected with SVV (MOI = 5), and samples were collected at indicated time and subjected to Western blot. Quantification analysis of VP3 expression with ImageJ, the level of VP3 band intensities normalized to that of β-actin. (**F**) HEK-293T cells seeded in six-well plates were transfected with indicated siRNAs and plasmids. At 36 hpt, the cells were infected with rSVV-eGFP (MOI = 0.5) for 12 h. Fluorescence of rSVV-eGFP was taken under a confocal microscope. (**G, H**) Viral infectivity was quantified by flow cytometry from the results of (**F**). The error bars indicate mean ± SD from three independent experiments (*, *P* < 0.05; ***, *P* < 0.001). (**I**) C2C12 cells were transduced with non-targeting sgRNA and TRIM32 targeting sgRNA, and the expression of TRIM32 was subjected to Western blot. (**J**) C2C12 cells transduced with non-targeting sgRNA and TRIM32 targeting sgRNA were infected with rSVV-eGFP (MOI = 0.5) for 12 h, and virus infectivity was measured by flow cytometry. (**K**) Viral infectivity was quantified by flow cytometry from the results of (**J**). The error bars indicate mean ± SD from three independent experiments (**, *P* < 0.01). (**L**) C2C12 cells transduced with non-targeting sgRNA and TRIM32 targeting sgRNA were infected with SVV (MOI = 0.5) for 12 h. The virus titers in the supernatants at 12 hpi were determined using the TCID_50_ assay. The error bars indicate mean ± SD from three independent experiments (**, *P* ＜0.01). (**M**) C2C12 cells transduced with non-targeting sgRNA and TRIM32 targeting sgRNA were infected with SVV (MOI = 0.5) for 12 h, and the expression of TRIM32 and VP3 was subjected to Western blot.

Examination of the impact of TRIM32 on SVV propagation showed that siRNA-mediated knockdown led to a significant reduction in the TRIM32 protein level (Fig. 2A), but silencing of TRIM32 significantly enhanced SVV infection, as reflected by a considerable increase in the viral titer (Fig. 2B) and VP3 production (Fig. 2D). Conversely, compared to the downregulation of TRIM32, the ectopic expression of TRIM32 severely inhibited SVV replication (Fig. 2C and E). To verify these findings, HEK-293T cells were infected with recombinant eGFP-expressing SVV (rSVV-eGFP), and siRNA-mediated knockdown of TRIM32 enhanced fluorescence signals (Fig. 2F and G), whereas the fluorescence signal was robustly decreased in TRIM32-expressing cells (Fig. 2F and H). CRISPR-Cas9 editing was utilized to knock down TRIM32 in C2C12 cells (Fig. 2I through K), and a moderate recovery of infection was detected in the TRIM32-deficient cells (Fig. 2J and K). Knockdown of TRIM32 enhanced SVV replication (Fig. 2L and M). Collectively, these results indicate that TRIM32 exhibits antiviral activity against SVV.

### SVV 3C^pro^ cleaves and degrades TRIM32

**Fig 3:**
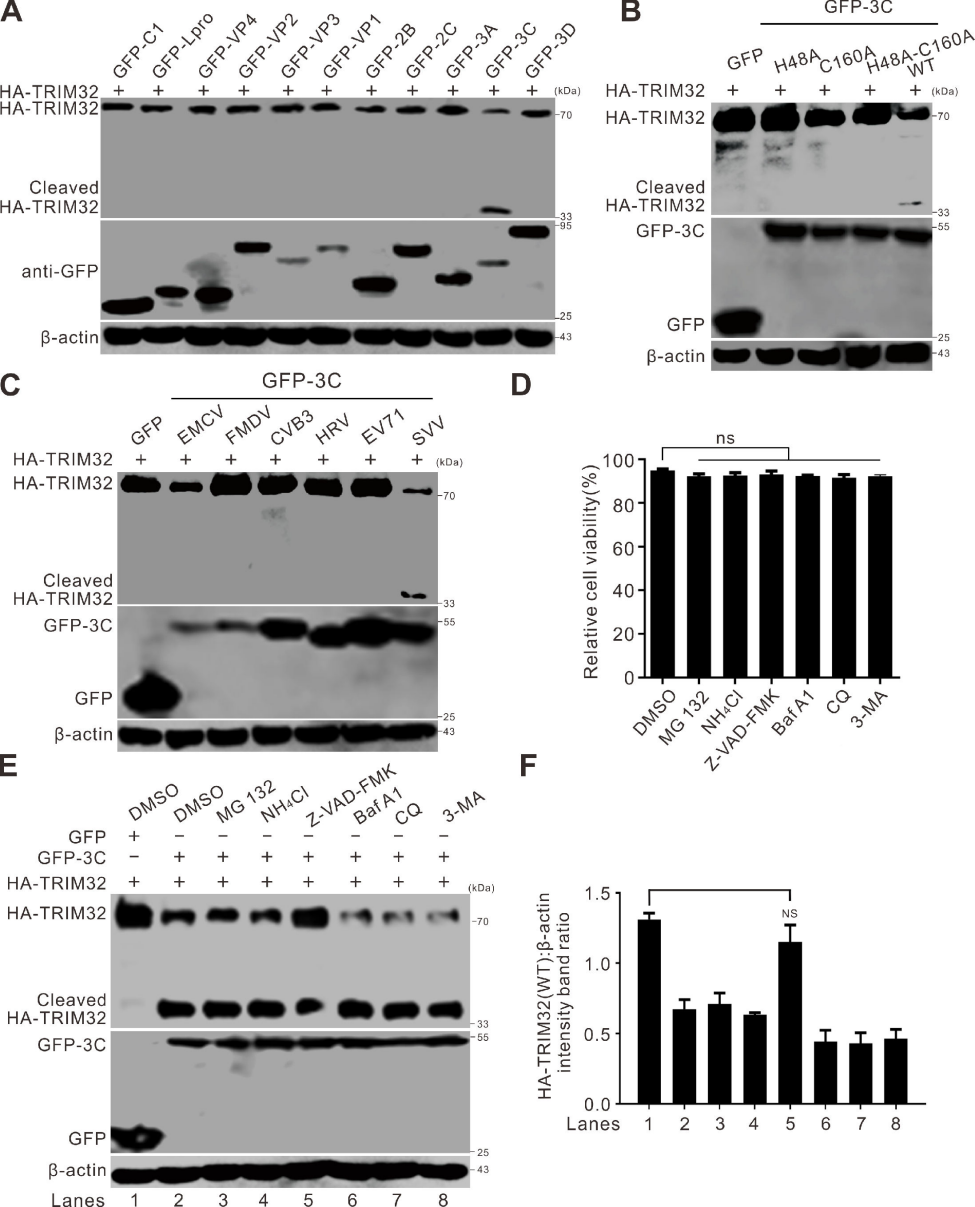
SVV 3C^pro^ cleaves TRIM32. (**A**) BHK-21 cells seeded in six-well plates were cotransfected with HA-TRIM32 and GFP-tagged SVV protein coding plasmids for 24 h. The expression of HA-TRIM32 was determined by Western blot. (**B**) BHK-21 cells in six-well plates were cotransfected with HA-TRIM32, GFP-3C, and its mutants (GFP-3C^H48A^, GFP-3C^C160A^, and GFP-3C^H48A-C160A^). The expression of HA-TRIM32 was detected by Western blot. (**C**) BHK-21 cells in six-well plates were cotransfected with HA-TRIM32, GFP-tagged 3C from EMCV, FMDV, CVB3, HRV, EV71, and SVV. The expression of HA-TRIM32 was determined by Western blot. (**D**) The viability of BHK-21 cells was examined using the CCK-8 assay after treatment with DMSO, MG132 (10 µM), NH_4_Cl (10 mM), Z-VAD-FMK (50 µM), bafilomycin A1 (Baf A1, 200 nM), chloroquine (CQ, 40 µM), and 3-methyladenine (3-MA, 25 mM). The error bars stand for standard deviation (SD) from three independent experiments (ns, not significant). (**E**) BHK-21 cells in six-well plates were cotransfected with HA-TRIM32, GFP-3C, or GFP vector for 20 h. The cells were treated with MG132 (10 µM), NH_4_Cl (10 mM), Z-VAD-FMK (50 µM), bafilomycin A1 (Baf A1, 200 nM), chloroquine (CQ, 40 µM), and 3-methyladenine (3-MA, 25 mM) for 12 h. The expression of HA-TRIM32 was determined by Western blot. (**F**) Quantification analysis of protein expression from (**E**) with ImageJ, and the level of full-length HA-Trim32 band ratios was normalized to β-actin.

To identify the viral proteins responsible for inducing the cleavage of TRIM32, BHK-21 cells were cotransfected with SVV protein expression plasmids and an HA-tagged TRIM32 construct. The expression of GFP-3C led to the appearance of two detectable bands (Fig. 3A). This observation was in line with the results obtained from SVV-infected cells (Fig. 1A through C), indicating that SVV 3C^pro^ was responsible for the cleavage of TRIM32. SVV 3C^pro^ with protease activity-deficient mutants, consisting of 3C^H48A^, 3 C^C160A^, and 3C^DM^ (H48A and C160A), which were used in our previous studies (10, 11), failed to cleave and degrade TRIM32 (Fig. 3B), suggesting that the abrogation of the catalytic sites in 3C^pro^ eliminates its cleavage and degradation activities towards TRIM32. We further examined 3C^pro^ from other *Picornaviruses*, including encephalomyocarditis virus (EMCV), foot-and-mouth disease virus (FMDV), coxsackievirus B3 (CVB3), human rhinovirus (HRV), and enterovirus 71 (EV71) (Fig. 3C). The results revealed that only SVV 3C^pro^ was capable of specifically cleaving TRIM32. Cellular toxicity was evaluated using the Cell Counting Kit 8 assay. The findings showed that the viability of cells treated with MG132 (10 µM), NH_4_Cl (10 mM), Z-VAD-FMK (50 µM), bafilomycin A1 (Baf A1, 200 nM), chloroquine (CQ, 40 µM), 3-methyladenine (3-MA, 25 mM) remained largely unchanged (Fig. 3D). Treatment with the pan-caspase inhibitor, Z-VAD-FMK, significantly alleviated the degradation of TRIM32, indicating that 3C^pro^-induced TRIM32 degradation was caspase-dependent (Fig. 3E and F).

### SVV 3C^pro^-cleaved TRIM32 at E332 loses its antiviral activity

**Fig 4:**
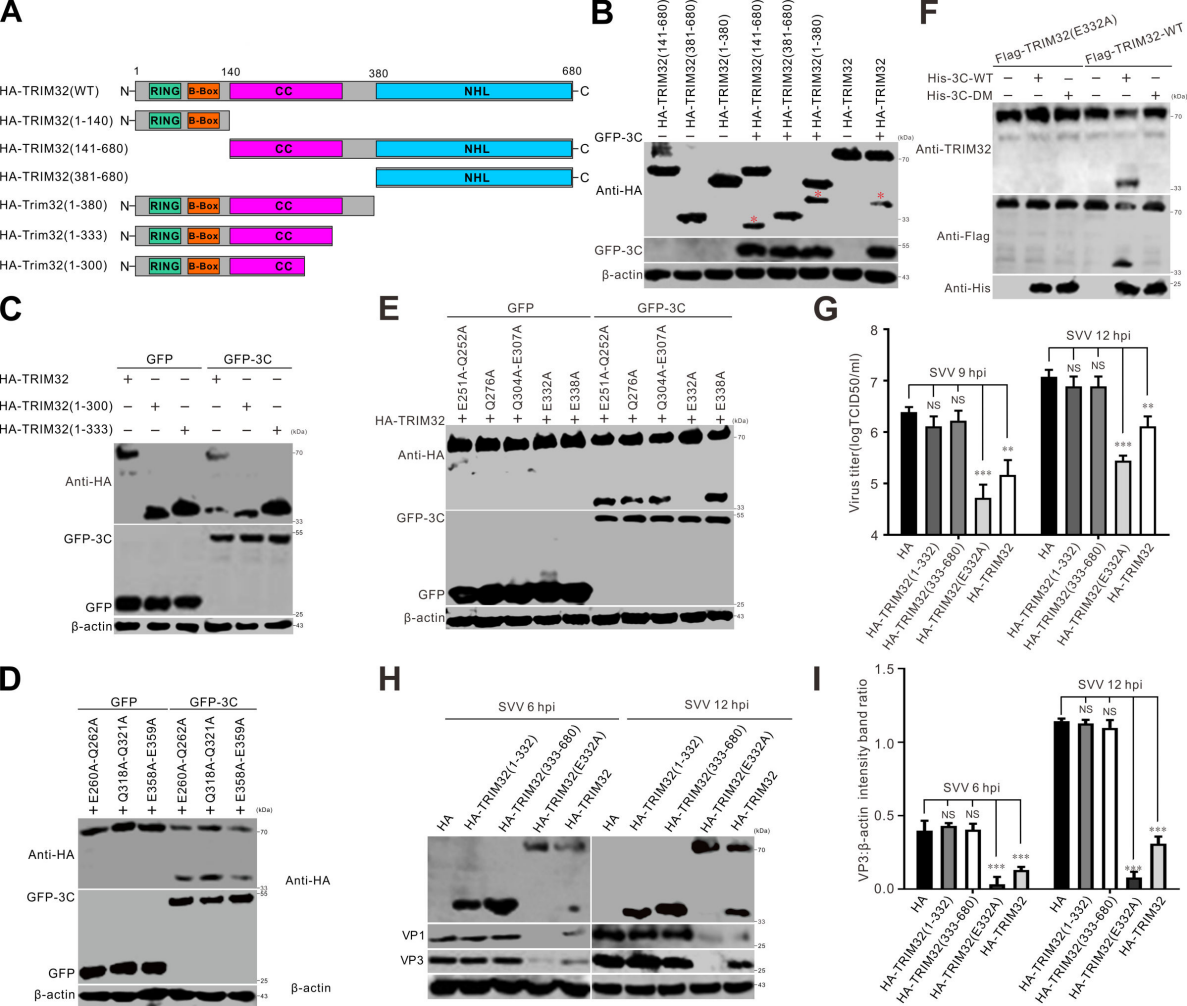
Cleaved TRIM32 loses its antiviral activity. (**A**) Schematic illustration of the structural domains and truncated TRIM mutants. (**B and C**) BHK-21 cells seeded in six-well plates were cotransfected with HA-TRIM32 and its truncation constructs with GFP-3C for 24 h, and the samples were analyzed by Western blot. (**D and E**) BHK-21 cells in six-well plates were cotransfected with HA-TRIM32 and its mutation constructs with GFP-3C for 24 h, and the samples were analyzed by Western blot. (**F**) *In vitro* cleavage assay of TRIM32. Anti-Flag resin-purified Flag-TRIM32 (5 µg) and Flag-TRIM32-E332 (5 µg) were incubated with purified His-3C-WT (10 µg) or His-3C-DM (10 µg) at 37°C for 2 h, followed by Western blot. (**G**) BHK-21 cells in six-well plates were cotransfected with HA vector, HA-TRIM32, and its truncation constructs for 24 h, then infected with SVV for 9 and 12 h. The virus titers in the supernatants were determined using the TCID_50_ assay. (**H**) BHK-21 cells in six-well plates were cotransfected with HA vector, HA-TRIM32, and its truncation constructs for 24 h, then infected with SVV for 6 and 12 h, and the samples were analyzed by Western blot. (**I**) Quantification presented with graphs representing VP3 normalized against β-actin using ImageJ from (**H**).

Picornavirus 3C^pro^ has a preference for targeting glutamine-glycine (Q-G) or glutamic acid-glutamine (E-Q) pairs of cellular proteins for cleavage (39). To explore the specificity of the cleavage site on TRIM32, several truncated plasmids were constructed based on the molecular weight of the cleaved TRIM32 product, including HA-TRIM32 (1-380), HA-TRIM32 (381-680), and HA-TRIM32 (141-680) (Fig. 4A). Western blot showed that the size of the cleaved N-terminal HA-TRIM32 product was slightly shorter than that of the TRIM32 (1-380) mutant (Fig. 4B); both TRIM32 (1-380) and TRIM32 (141-680) were cleaved by GFP-3C, generating two fragments (Fig. 4B), indicating that the potential cleavage site of TRIM32 was located between residues 141 and 380. Construction of HA-TRIM32 (1-300) and HA-TRIM32 (1-333) indicated that the cleavage site was near aa.300-333 (Fig. 4C). When site-directed mutations were introduced at Q or E residues in TRIM32, potentially acting as cleavage sites, TRIM32 (E260A-Q262A), TRIM32 (Q318A-Q321A), and TRIM32 (E358A-E359A) were still cleaved by 3C^pro^ (Fig. 4D). However, the E332 mutation in TRIM32 was resistant to 3C-mediated cleavage, confirming that E332 was recognized by SVV 3C^pro^ for cleaving TRIM32 (Fig. 4E). Moreover, we conducted an *in vitro* cleavage assay to verify if TRIM32 can be directly cleaved by SVV 3C^pro^. SVV 3 C-WT (wild-type) and 3 C-DM (double mutation) were expressed and purified from *Escherichia coli*. Flag-TRIM32-WT and Flag-TRIM32-E332 were purified from BHK-21 cells using anti-Flag beads. Subsequently, the *in vitro* cleavage assay was carried out as previously described (8, 40). As shown in Fig. 4F, two cleaved bands were observed for Flag-TRIM32-WT in the presence of 3 C-WT but not 3 C-DM. However, the Flag-TRIM32-E332 mutant resisted 3C^pro^-mediated cleavage. Overexpression of TRIM32-WT and TRIM32-E332A significantly reduced SVV viral titers in BHK-21 cells (Fig. 4G). In contrast, cleaved TRIM32 (1-332) and TRIM32 (333-680) lost their antiviral activity (Fig. 4G), suggesting that cleavage of TRIM32 abolished its antiviral activity. In terms of the effects of TRIM32 cleavage products and their cleavage sites on SVV VP3 production (Fig. 4H and I), compared with TRIM32-WT, TRIM32 (1-332) and TRIM32 (333-680) lost their inhibitory effects against SVV infection (Fig. 4H and I), whereas the cleavage site mutant TRIM32-E332A enhanced its ability to suppress SVV replication and VP3 production (Fig. 4H and I), indicating that the 3C cleavage-resistant TRIM32 mutant had stronger antiviral effects than TRIM32-WT. Together, these data indicate that 3C^pro^-mediated cleavage of TRIM32 at E332 and the cleaved products failed to inhibit SVV infection.

### TRIM32 targets the viral VP3 protein for proteasomal degradation

**Fig 5:**
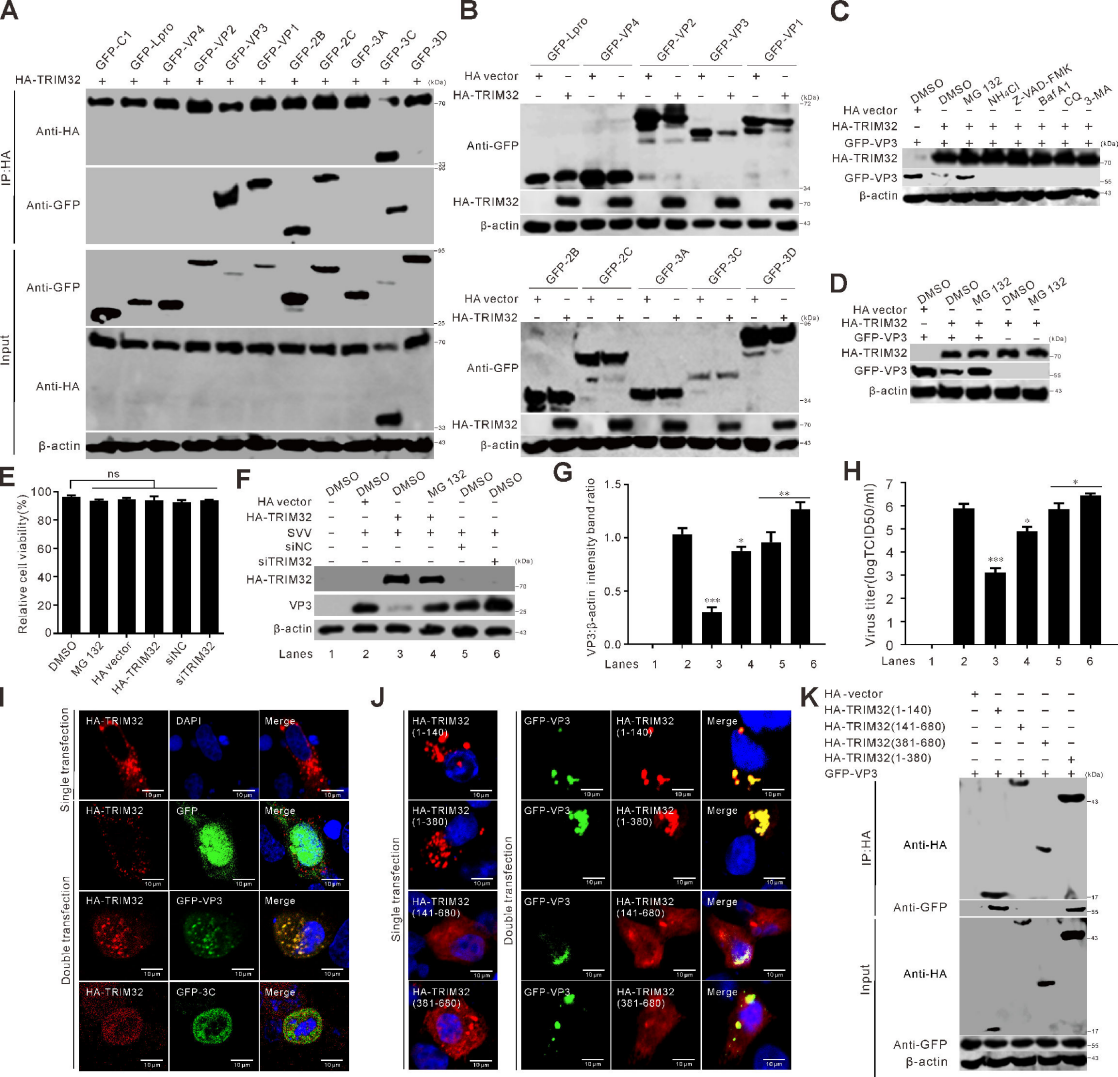
TRIM32 targets VP3 for degradation. (**A**) BHK-21 cells seeded in six-well plates were cotransfected with HA-TRIM32 and GFP-tagged SVV protein coding plasmids for 24 h. Co-IP was performed using anti-HA magnetic beads. Precipitated proteins were analyzed by Western blot. (**B**) BHK-21 cells in six-well plates were cotransfected with HA vector or HA-TRIM32 with GFP-tagged plasmids encoding SVV proteins for 24 h. The expression of GFP-tagged plasmids was determined by Western blot. (**C**) BHK-21 cells in six-well plates were cotransfected with HA vector or HA-TRIM32 with GFP-VP3 for 20 h. The cells were treated with MG132 (10 µM), NH_4_Cl (10 mM), Z-VAD-FMK (50 µM), Baf A1 (200 nM), CQ (40 µM), and 3-MA (25 mM) for 12 h. The expression of GFP-VP3 was determined by Western blot. (**D**) BHK-21 cells in six-well plates were cotransfected with HA vector or HA-TRIM32 with GFP-VP3 for 20 h. The cells were treated with MG132 (10 µM) for 12 h. The expression of GFP-VP3 was determined by Western blot. (**E**) The viability of PK-15 cells was examined using the CCK-8 assay after treatment with DMSO, MG132 (10 µM), and transfected with HA-Trim32, HA empty vector, siNC, or siTrim32. The error bars stand for standard deviation (SD) from three independent experiments (ns, not significant). (**F**) PK-15 cells in six-well plates were cotransfected with HA vector or HA-TRIM32, and siNC or siRNA targeting TRIM32 for 24 h. The cells were infected with SVV and treated with MG132 (10 µM) for 12 h. The expression of VP3 was determined by Western blot. (**G**) Quantification analysis of protein expression from (**F**) with ImageJ, the level of VP3 band ratios was normalized to β-actin. (**H**) PK-15 cells in six-well plates were cotransfected with HA vector or HA-TRIM32, and siNC or siRNA targeting TRIM32 for 24 h. The cells were infected with SVV (MOI = 5) and treated with MG132 (10 µM) for 12 h. The virus titers in the supernatants were determined using the TCID_50_ assay. (**I and J**) Fluorescence analysis was performed to analyze the transfection of plasmids expressing indicated GFP-tagged (green), HA-TRIM32 (red), and its truncation constructs, which were stained with the HA-tagged antibody (red), and DAPI (blue) in BHK-21 cells, and then taken under a confocal microscopy. (**K**) BHK-21 cells in six-well plates were cotransfected with HA-TRIM32 truncation constructs and GFP-VP3 for 24 h. Co-IP was performed using anti-HA magnetic beads. Precipitated proteins were analyzed by Western blot.

Co-IP assays were conducted to explore the interactions between TRIM32 and SVV structural and nonstructural proteins. The results revealed that VP3, VP1, 2B, 2C, and 3C interacted with TRIM32 (Fig. 5A). Overexpression of TRIM32 resulted in significant degradation of VP3 but not of other viral proteins (Fig. 5B). Moreover, in the presence of MG132, a proteasome inhibitor, TRIM32-induced VP3 degradation was restored, but not by NH_4_Cl, Z-VAD-FMK, BafA1, CQ, or 3-MA (Fig. 5C). MG132 treatment has no impact on the expression levels of TRIM32 (Fig. 5D), and MG132 addition promoted SVV infection in TRIM32-expressing PK-15 cells (Fig. 5F through H). There were no significant impacts on the viability of PK-15 cells that were treated with MG132 (10 µM) and transfected with HA-Trim32, HA empty vector, siNC, or siTrim32 (Fig. 5E). Consistent with the results of Co-IP, confocal microscopy indicated that VP3 colocalized with TRIM32 and exhibited puncta distribution in the cytoplasm, whereas 3C disrupted the puncta structure (Fig. 5I). These results suggest that TRIM32 selectively targets SVV VP3 for proteasomal degradation, which is an important part of its antiviral mechanism. SVV 3C^pro^ cleaved TRIM32 at residue E332, separating the RING and NHL domains (Fig. 4A). Compared to TRIM32-WT, TRIM32 (1–140) and TRIM32 (1–380) contain the N-terminal RING domain, exhibit similar cellular puncta distribution patterns, and are colocalized with VP3 in the cytoplasm, whereas TRIM32 (141–680) and TRIM32 (381––680), lacking the N-terminal RING domain, are localized in the nucleus and cytoplasm (Fig. 5J). These colocalization results were in line with the Co-IP assay results. The fragments TRIM32 (1–140) and TRIM32 (1–380), which possess the RING domain, interacted with VP3 (Fig. 5K). Collectively, these results suggest that TRIM32 suppresses SVV replication by degrading VP3 in a proteasome-dependent manner.

### Cleaved TRIM32 has impaired E3 ubiquitin ligase activity

**Fig 6:**
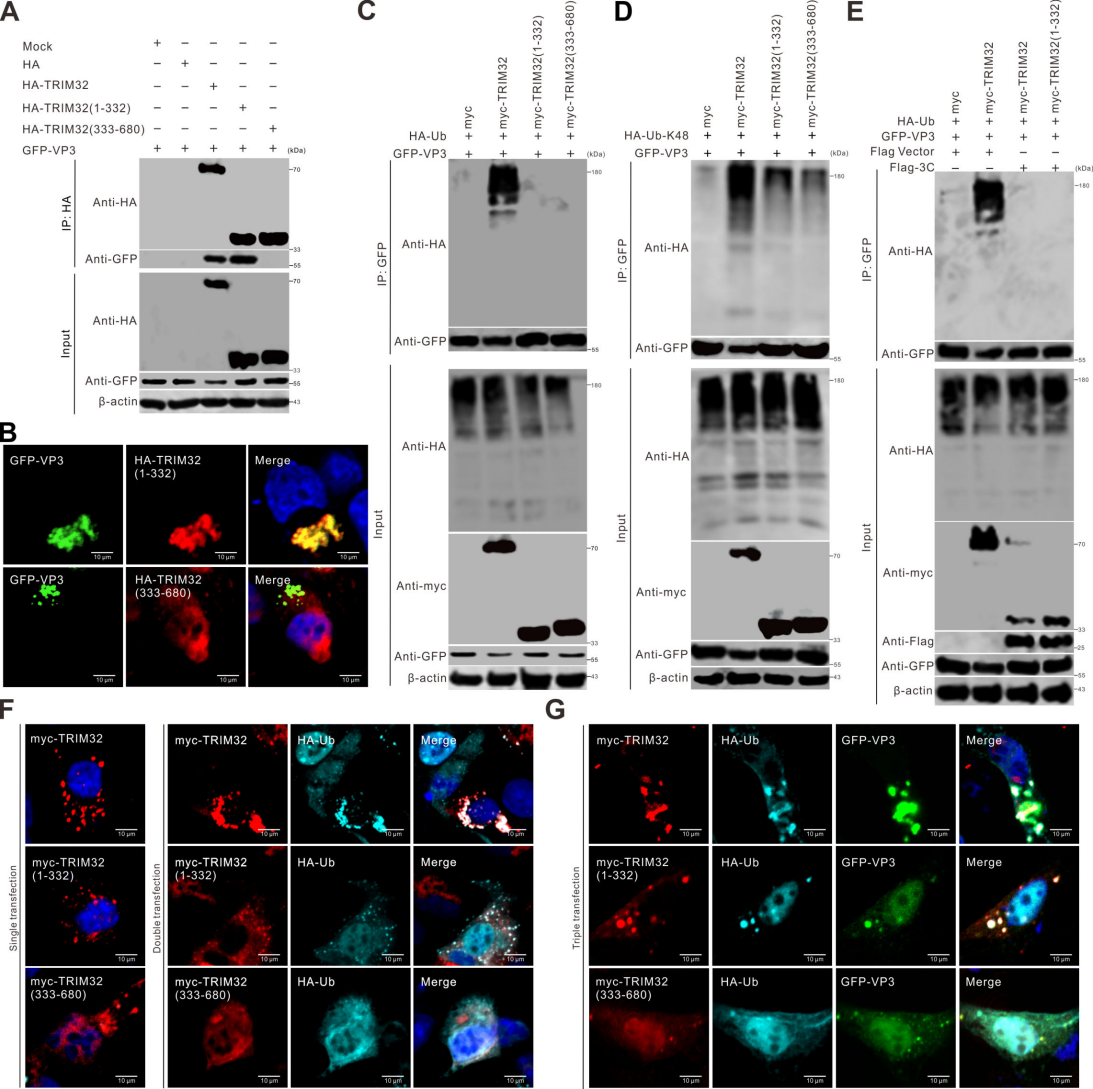
Cleaved TRIM32 has impaired E3 ubiquitin ligase activity. (**A**) BHK-21 cells seeded in six-well plates were cotransfected with HA-TRIM32 and its truncation constructs with GFP-VP3 for 24 h. Co-IP was performed using anti-HA magnetic beads. Precipitated proteins were analyzed by Western blot. (**B**) Fluorescence microscopy was utilized to analyze the transfection of plasmids expressing indicated GFP-VP3 (green), HA-TRIM32 (red), and its truncation constructs, which were stained with the HA-tagged antibody (red), and DAPI (blue) in BHK-21 cells, and then taken under a confocal microscopy. (**C**) BHK-21 cells in six-well plates were cotransfected with myc-TRIM32 and its truncation construct, HA-Ub (ubiquitin) with GFP-VP3 for 24 h. Co-IP was performed using anti-GFP magnetic beads. Precipitated proteins were analyzed by Western blot. (**D**) BHK-21 cells in six-well plates were cotransfected with myc-TRIM32 and its truncation construct, HA-Ub-K48 with GFP-VP3 for 24 h. Co-IP was performed using anti-GFP magnetic beads. Precipitated proteins were analyzed by Western blot. (**E**) BHK-21 cells in six-well plates were cotransfected with myc-TRIM32 and its truncation construct, HA-Ub, Flag-3C, or Flag empty vector with GFP-VP3 for 24 h. Co-IP was performed using anti-GFP magnetic beads. Precipitated proteins were analyzed by Western blot. (**F, G**) Fluorescence microscopy was employed to analyze the transfection of plasmids expressing indicated GFP-VP3 (green), myc-TRIM32 (red), and its truncation constructs (red), which were stained with myc-tagged antibody, HA-Ub (cyan), and DAPI (blue) in BHK-21 cells, and then taken under a confocal microscopy.

Compared to TRIM32-WT, TRIM32 (1–332) showed an interaction with VP3, whereas the interactions between TRIM32 (333–680) and VP3 were abolished (Fig. 6A). TRIM32 (1–332) harbors an N-terminal RING domain and was observed colocalized with VP3 within cells (Fig. 6B), indicating that TRIM32 degrades VP3 depending on its RING domain. Ubiquitination plays a central role in proteasome-dependent proteolytic pathways. TRIM32 is widely recognized to possess E3 ubiquitin ligase activity due to the presence of its N-terminal RING finger domain, a conserved structural motif mediating the ubiquitination of target proteins (41). SVV 3C^pro^ specifically cleaved TRIM32 at the E332 residue, resulting in the physical separation of the RING domain and NHL domain (Fig. 4A). Therefore, we hypothesized that cleaved TRIM32 products could potentially disrupt E3 ubiquitin ligase activity. VP3 immunoprecipitated ubiquitin in the presence of TRIM32 (Fig. 6C) and cotransfection with TRIM32 increased the levels of VP3 ubiquitination, suggesting that TRIM32 mediates the ubiquitination of VP3, leading to the degradation of VP3 by the proteasome, thereby contributing to its antiviral role. In *in vitro* ubiquitination assays, co-expression of VP3 and HA-ubiquitin revealed that TRIM32-WT specifically promoted VP3 polyubiquitination, whereas its two cleavage fragments failed to exhibit this activity (Fig. 6C). Further study identified that TRIM32 induces K48-linked polyubiquitination of VP3, a modification absent when using the two cleaved products (Fig. 6D). Importantly, SVV 3C^pro^ counteracted TRIM32-mediated downregulation of VP3 expression and the ubiquitination modification of the VP3 protein induced by TRIM32 (Fig. 6E). TRIM32-WT and HA-ubiquitin formed aggregates in the cytoplasm; TRIM32 (1–332), which contains the RING domain, exhibited a reduced colocalization ratio, while TRIM32 (333–680) failed to form aggregates (Fig. 6F). Additionally, we observed punctate cytoplasmic colocalization of TRIM32-WT with ubiquitin and VP3 in the cytoplasm (Fig. 6G). In contrast, the truncated TRIM32 fragments displayed significantly reduced colocalization efficiency with ubiquitin and VP3 (Fig. 6G). These results demonstrated that the cleaved TRIM32 products markedly weaken their ability to conjugate ubiquitin to VP3 for proteasome-dependent degradation. Taken together, these results revealed that the degradation of VP3 by TRIM32 is involved in the ubiquitination pathway, and the cleavage of TRIM32 impairs its E3 ubiquitin ligase activity.

### Cleaved TRIM32 has impaired type I IFN induction

**Fig 7:**
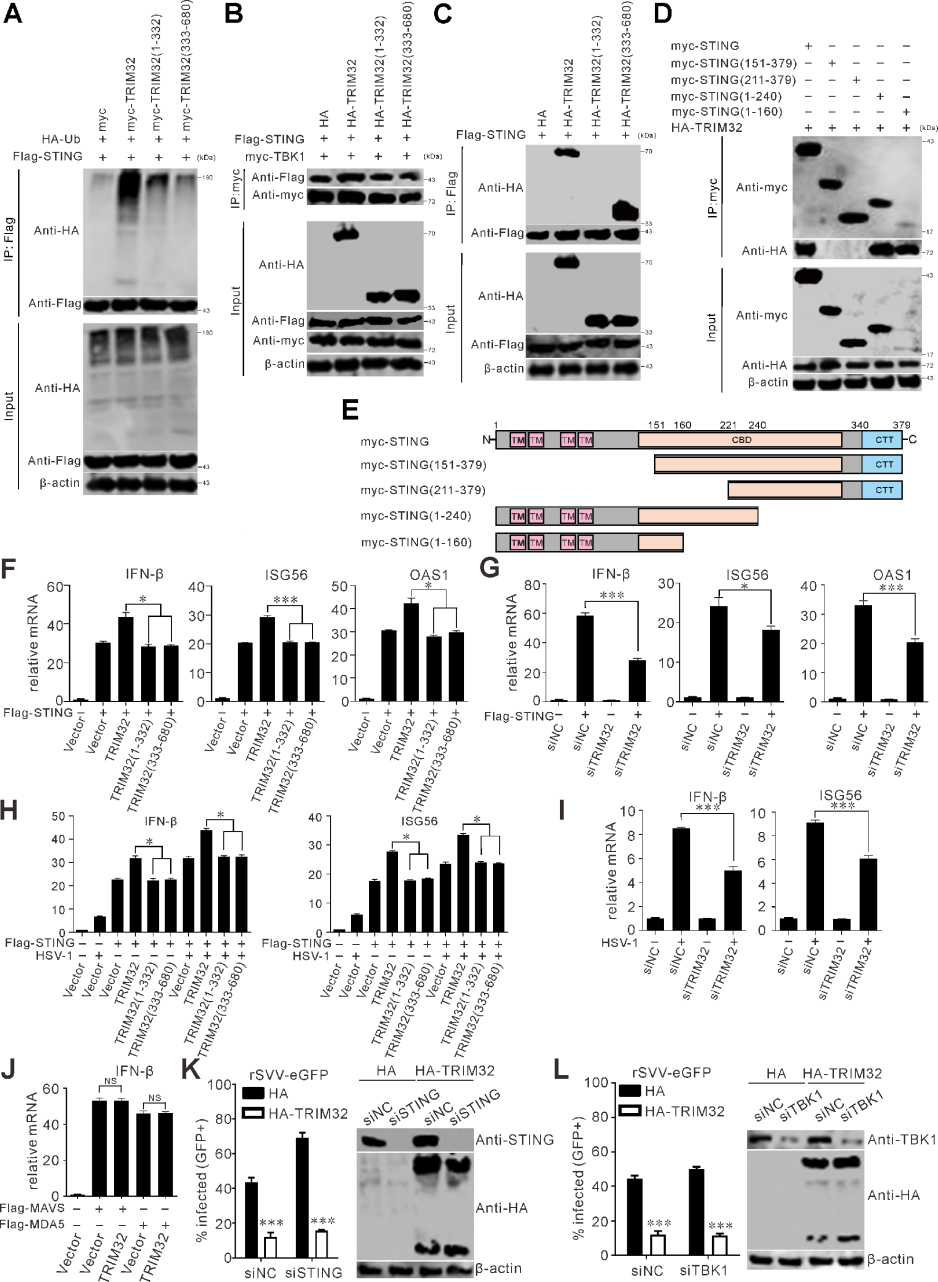
Cleaved TRIM32 displays reduced capacity to induce type I interferon. (**A**) HEK-293T cells were cotransfected with myc-TRIM32 and its truncation construct, along with Flag-STING and HA-Ub for 24 h. Co-IP was performed using anti-Flag magnetic beads. Precipitated proteins were analyzed by Western blot. (**B–D**) BHK-21 cells seeded in six-well plates were cotransfected with indicated plasmids for 24 h. Co-IP was performed using anti-myc or anti-Flag magnetic beads. Precipitated proteins were analyzed by Western blot. (**E**) Schematic diagram of the structural domains and truncated STING mutants. (**F**) HEK-293T cells transfected with myc-TRIM32 and its truncation construct for 24 h. The transcriptional expression level of IFN-β, ISG56, and OAS1 was analyzed by RT-qPCR and normalized to GADPH mRNA. Error bars indicate mean ± SD from three independent infection experiments. (**G**) HEK-293T cells transfected with siNC or siTrim32 and Flag-STING for 24 h. The transcriptional expression levels of IFN-β, ISG56, and OAS1 were analyzed using quantitative RT-PCR and normalized to GAPDH mRNA. Error bars indicate the mean ± SD from three independent infection experiments. (**H**) HEK-293T cells transfected myc-TRIM32 and its truncation constructs and infected with HSV-1 (MOI = 0.1) for 24 h. The transcriptional expression levels of IFN-β and ISG56 were analyzed using quantitative RT-PCR and normalized to GAPDH mRNA. Error bars indicate the mean ± SD from three independent infection experiments. (**I**) HEK-293T cells transfected with siNC or siTrim32 and infected with HSV-1 (MOI = 0.1) for 24 h. The transcriptional expression levels of IFN-β and ISG56 were analyzed using quantitative RT-PCR and normalized to GAPDH mRNA. Error bars indicate the mean ± SD from three independent infection experiments. (**J**) HEK-293T cells were cotransfected myc-TRIM32 with Flag-MAVS, Flag-MDA5, or Flag empty vector for 24 h. The transcriptional expression levels of IFN-β were analyzed using quantitative RT-PCR and normalized to GAPDH mRNA. Error bars indicate the mean ± SD from three independent infection experiments. (**K**) HeLa cells transfected with siNC or siSTING with or without HA-TRIM32 expression were infected with rSVV-eGFP (MOI = 0.1) for 12 h, and viral infectivity was quantified using flow cytometry. Western blot analysis was conducted to assess the siRNA-mediated reduction of STING expression using a specific antibody targeting STING. (**L**) HeLa cells transfected with siNC or siTBK1 with or without HA-TRIM32 expression were infected with rSVV-eGFP (MOI = 0.1) for 12 h, and viral infectivity was quantified using flow cytometry. Western blot analysis was conducted to assess the siRNA-mediated reduction of TBK1 expression using a specific antibody targeting TBK1.

TRIM32 has been shown to target STING for K63-linked ubiquitination, which positively regulates the type I IFN pathway (32). Our findings indicate that TRIM32 effectively induces STING ubiquitination, while TRIM32 (1–332) only slightly promotes this ubiquitination event (Fig. 7A). In contrast, TRIM32 (333––680), which lacks the RING domain, completely loses its ability to ubiquitinate STING (Fig. 7A). Previous studies have demonstrated that TRIM32 is necessary for interaction between STING and TBK1 (32). TRIM32-WT, but not the cleaved fragments TRIM32 (1–332) or TRIM32 (333–680), notably enhanced the interaction between STING and TBK1 (Fig. 7B). TRIM32 interacted with STING, TRIM32 (333–680) containing the NHL domain, interacts specifically with the transmembrane domain (TMD) of STING (Fig. 7C through E). Overexpression of TRIM32 positively regulated the IFN-I immune response in the presence of STING, as reflected by upregulated transcription IFN-β, ISG56, and OAS1 mRNA (Fig. 7F). In contrast, the cleaved products of TRIM32 lost the regulatory effects on inducing the expression of type I IFN signaling molecules and ISGs (Fig. 7F). Consistently, knockdown of TRIM32 attenuated STING-induced type I IFN signaling and transcription of ISGs, including IFN-β, ISG56, and OAS1 (Fig. 7G). Moreover, following HSV-1 (herpes simplex virus type 1) infection, TRIM32 played a positive regulatory role in the IFN-I immune response, but not the cleaved products, as evidenced by the increased transcription of IFN-β and ISG56 (Fig. 7H). Depletion of TRIM32 weakened HSV-1-mediated IFN-I signaling and reduced the transcription of ISG56 (Fig. 7I). Myc-TRIM32 was cotransfected with Flag-MAVS (mitochondrial antiviral signaling), Flag-MDA5 (melanoma differentiation-associated protein 5), or a Flag empty vector into HEK-293T cells for 24 h. Subsequently, quantitative RT-PCR was employed to analyze the transcriptional expression levels of IFN-β. The findings demonstrated that in the presence of TRIM32, overexpressing MAVS or MDA5 does not result in an increase in the transcriptional level of IFN - β (Fig. 7J). These results suggest that TRIM32-mediated antiviral activity occurs independently of the canonical MAVS signaling axis. Next, we aimed to ascertain whether TRIM32 inhibits SVV replication by regulating STING or through other mechanisms. We employed siRNA-mediated knockdown to silence STING in HeLa cells. Subsequently, we tested the impact of TRIM32 on SVV infection in the absence of STING (Fig. 7K). Significantly, even when STING was depleted in HeLa cells, TRIM32 still maintained its anti-SVV activity (Fig. 7I). In addition, we silenced TBK1 in TRIM32-expressing cells. Again, SVV infection was strikingly reduced by TRIM32 expression in the absence of TBK1 (Fig. 7L). Overall, our results show that TRIM32 suppresses SVV replication without relying on the typical IFN-mediated antiviral pathways. Taken together, these results reveal that TRIM32 promotes IFN-β production through the induction of STING ubiquitination and the enhancement of STING-TBK1 interaction, and SVV 3C^pro^-mediated cleavage of TRIM32 weakens its ability to regulate IFN-β production.

## DISCUSSION

Recently, an increasing number of mammalian TRIM proteins have been identified as crucial immunoregulators of innate immunity, playing a vital role in defense against viral infection (42–48). In the present study, we identified TRIM32 as a host restriction factor capable of inhibiting SVV replication. TRIM32 directly restricts SVV replication by targeting viral VP3 for proteasomal degradation. SVV 3C^pro^-mediated TRIM32 cleavage products reduced the capacity to induce VP3 proteasomal degradation and diminish its ability to inhibit SVV replication.

TRIM proteins are emerging as significant cell-intrinsic antiviral factors (44). These proteins exert antiviral effects through at least three principal mechanisms: degrading specific viral components, inducing IFN and proinflammatory cytokines, and modulating autophagy pathways (17). For instance, TRIM79a and TRIM52 play significant roles in targeting flavivirus nonstructural proteins for degradation; these two proteins act as crucial elements in the defense mechanism against flaviviruses (23, 49). Extensive studies have revealed that TRIM proteins are actively engaged in the fight against IAVs. TRIM22, TRIM41, TRIM14, and TRIM32 exhibit their inhibitory effects on IAV by degrading viral nucleoprotein (NP) or polymerase basic protein 1 (PB1) (29, 50–52). TRIM14 mediates the proteasome-dependent degradation of NP and effectively blocks the translocation of NP from the cytoplasm to the nucleus, thereby further impeding the reproduction of IAV (51). TRIM33 and the non-TRIM E3 ligase C-terminus of Hsc70-interacting protein (CHIP) also contribute to the inhibition of human immunodeficiency virus type 1 (HIV-1) replication. TRIM33 achieves this by degrading viral integrase, whereas CHIP targets the viral Tat protein for degradation (53, 54). Moreover, TRIM5α is a crucial effector in the IFN-induced inhibition of HIV-1 infection (48). These examples illustrate the diverse strategies employed by different proteins to combat various viruses.

The overexpression of TRIM32 inhibited SVV replication, whereas TRIM32 deficiency promoted SVV infection (Fig. 2). SVV 3C^pro^-mediated cleavage products, TRIM32 (1–332) and TRIM32 (333–680), abolished its antiviral activity (Fig. 4G through I). These two cleaved fragments of TRIM32 were unable to degrade VP3 (Fig. 6A), resulting in failing to inhibit SVV infection (Fig. 4G through I). Previous studies revealed that SVV 3C^pro^ antagonizes host antiviral factors by inducing the cleavage and degradation of DHX30 (55), indicating that SVV has developed numerous strategies to neutralize host antiviral factors by targeting cleavage, thereby abolishing their antiviral effects.

The TRIM protein superfamily currently encompasses over 100 members, which are characterized by tripartite motifs, including the N-terminal RING domain, B-box, and coiled coil (CC) region (56), and a variable C-terminus, such as a PRY/SPRY domain, or the NHL domain (57). For the majority of TRIM family members, the RING domain is necessary for their capacity to restrict viral replication or modulate innate immunity (56). TRIM32 is an extremely versatile E3 ligase that participates in monoubiquitination and the formation of K48-linked or K-63-linked polyubiquitin chains that are covalently bound to target proteins (32). We found that SVV 3C^pro^ targeted TRIM32 for cleavage at glutamic acid 332 (E332), generating two cleaved TRIM32 fragments: N-terminal TRIM32 (1–332) and C-terminal TRIM32 (333–680) (Fig. 4A). N-terminal TRIM32 (1–332) consists of the RING domain, B-box, and CC region, while the C-terminal TRIM32 (333–680) contains the NHL domain (Fig. 4A). The two cleaved TRIM32 proteins lost their ability to inhibit SVV replication because the degradation of VP3 by TRIM32 was blocked (Fig. 6A). Although the N-terminal cleavage fragment containing the RING domain could still interact with VP3, it was unable to trigger VP3 degradation (Fig. 6A). Since ubiquitination is a crucial step in the proteasome-dependent degradation pathway, we examined whether VP3 ubiquitination contributed to the antiviral effects of TRIM32. Overexpression of TRIM32 enhanced the ubiquitination of VP3, indicating that TRIM32 mediated VP3 ubiquitination (Fig. 6C). The cleaved TRIM32 products dampened the E3 ubiquitin ligase activity, as reflected by decreased VP3 ubiquitination in the presence of cleaved TRIM32 products (Fig. 6C). TRIM7 functions as an antiviral effector that restricts multiple human enteroviruses. Specifically, TRIM7 targets the 2BC protein of the CVB3 for ubiquitination and degradation (21). CVB3 3C^pro^ cleaves TRIM7 to weaken its antiviral effects, resulting in impairment of E3 ubiquitin ligase activity (20). Our results show that some members of the *Picornaviridae* family adopt the same antagonistic action against TRIMs through cleavage by viral 3C^pro^. Interestingly, evolutionary pressure imposed by host restriction factor TRIM7 has driven the emergence of TRIM7-resistant coxsackievirus variants that have a single-point mutation in the viral 2C protein that contributes to evading TRIM7 but hampers viral replication (21). This provides a mechanistic framework for understanding the evolutionary trade-offs in viral fitness imposed by host restriction factors. Specifically, CVB3 3C^pro^ cleaves TRIM7 at Q24, and Q24 is crucial for optimal antiviral activity against CVB3 (20). E332A in TRIM32 was resistant to cleavage mediated by 3C^pro^, and TRIM32 (E332A) showed greater antiviral activity than TRIM32-WT (Fig. 4G and H). CVB3 3C^pro^ targets TRIM7 (Q24) for cleavage, generating 24 N-terminal residues and a C-terminal fragment containing the RING and PRY/SPRY domains. The 24 amino acids of TRIM7 are necessary for the degradation of viral 2BC but are not required for interaction with 2BC (20). A TRIM32 (1–332) fragment containing the RING domain is required for interaction with viral VP3 but is not necessary for the degradation of viral VP3 (Fig. 6A). Recent studies have uncovered distinct antiviral mechanisms employed by TRIM family members against RNA viruses. Specifically, TRIM56 exerts antiviral activity against Coxsackievirus B replication by ubiquitinating the viral 3D protein (58). In contrast, TRIM72 mediates proteasomal degradation of rabies virus matrix (M) protein through its E3 ligase activity (59). Meanwhile, TRIM32 restricts Venezuelan equine encephalitis virus infection at the post-entry stage by targeting the viral nucleocapsid protein for proteasomal degradation (60).

TRIM proteins serve as key elements of the innate immune system. They function either as antiviral restriction factors or as modulators of signaling cascades, which in turn trigger the induction of proinflammatory cytokines (45–47, 56). For example, TRIM5 inhibits SVV replication by enhancing the RIG-I-mediated IFN-I signaling (61). TRIM23 mediates virus-induced autophagy through the activation of TBK1, a process that is crucial for the autophagy-mediated restriction of multiple viruses (62). TRIM5α has been demonstrated to act as both a regulator of autophagy and an autophagic cargo receptor, facilitating the autophagic degradation of viral capsid proteins (63). TRIM32 serves as a key positive regulatory factor for IFN-β induction by promoting K63-linked polyubiquitination of STING, and silencing of TRIM32 led to a decreased level of IFN-β and suppressed the activation of IFN regulatory factor 3 (IRF3), indicating that TRIM32 demonstrates a broad spectrum of antiviral effects (64). HIV-1 p6 protein disrupts the interaction between STING and TRIM32 and negatively regulates the K27- and K63-linked ubiquitination of STING mediated by TRIM32 (65). TRIM32 significantly promotes STING ubiquitination, and the cleaved TRIM32 products, TRIM32 (1–332) and TRIM32 (333-680), lost their ability to ubiquitinate STING (Fig. 7A). TRIM32 is indispensable for the interaction between STING and TBK1, and the N-terminal TMD of STING and the C-terminal NHL domain of TRIM32 are crucial for their interaction (32). TRIM32-WT significantly strengthened the interaction between STING and TBK1, while the cleaved TRIM32 products do not (Fig. 7B), indicating that the ubiquitination of STING mediated by TRIM32 is necessary for the activation of downstream TBK1 and the production of IFN-I. The N-terminal TMD of STING and the C-terminal cleaved product, TRIM32 (333-680) containing the NHL domain, are essential for their interaction (Fig. 7C and D). TRIM32 has also been shown to reduce the replication of spring viremia of carp virus, infectious hematopoietic necrosis virus, and white spot syndrome virus, suggesting that TRIM32 could be a potential immune enhancer of fish hosts for viral infection (66–68). TRIM32 plays a critical role in virus-induced IFN-β and TNF-α production by mediating transcriptional activation of NF-κB and the IFN-β promoter (32). Compared with TRIM32-WT, the cleaved products of TRIM32 failed to activate IFN-β promoter and induce the expression of IFN-β and OAS1 (Fig. 7F and G). Correspondingly, TRIM32 depletion severely impaired STING-mediated type I IFN signaling and the transcription of ISG (Fig. 7G). These results indicate that TRIM32 promotes IFN production by ubiquitinating STING and strengthening the STING-TBK1 interaction, thereby facilitating the host’s antiviral innate immune response. In order to counteract these effects, SVV 3C^pro^ cleaves TRIM32, acting as an antagonist to TRIM32-mediated STING-TBK1 signaling and IFN-β production.

**Fig 8:**
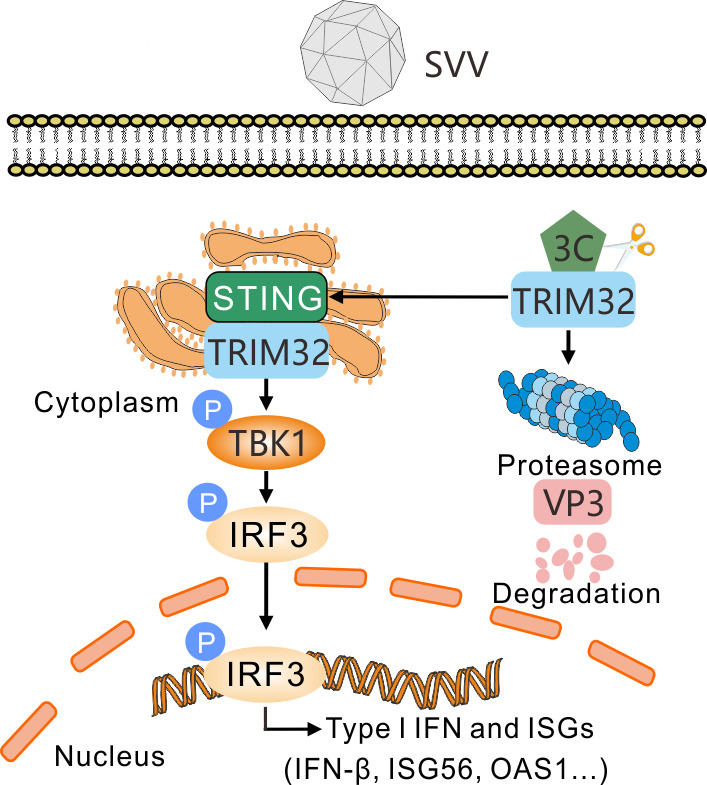
Proposed model for SVV restriction by the antiviral effects of TRIM32. TRIM32 restricts SVV replication that is degraded and cleaved during virus infection. Specifically, SVV 3C^pro^ induces the degradation and cleavage of TRIM32. TRIM32 restricts SVV replication by selectively targeting viral VP3 protein for ubiquitination and proteasomal degradation. 3C^pro^-mediated the cleavage products of TRIM32 lost their antiviral activity. Additionally, the cleaved TRIM32 products attenuate its function in the ubiquitination and degradation of viral VP3, as well as in the type I IFN signaling pathway. The study shows that SVV employs diverse strategies to evade the host’s innate immune responses.

In summary, TRIM32 exerts antiviral effects against SVV replication by targeting the viral VP3 protein for proteasomal degradation. Conversely, SVV 3C^pro^ cleaves TRIM32, thereby impairing both its proteasomal degradation function and type I IFN signaling pathway (Fig. 8). Taken together, these findings provide novel insights into the molecular mechanisms by which SVV counteracts the host’s intrinsic antiviral defense.

## Data Availability

The data that support the findings of this study are available from the corresponding author upon reasonable request.
